# A comprehensive practical review of acupoint embedding as a semi-permanent acupuncture: A mini review

**DOI:** 10.1097/MD.0000000000038314

**Published:** 2024-06-07

**Authors:** Amir Hooman Kazemi, Mohammad Sadegh Adel-Mehraban, Moein Jamali Dastjerdi, Reihane Alipour

**Affiliations:** a Department of Traditional Medicine, School of Persian Medicine, Tehran University of Medical Sciences, Tehran, Iran; b International School, Beijing University of Chinese Medicine, Beijing, China.; c Traditional Persian Medicine and Complementary Medicine (PerCoMed) Student Association, Students’ Scientific Research Center, Tehran University of Medical Sciences, Tehran, Iran; d Department of Traditional Medicine, School of Traditional Medicine, Shahid Beheshti University of Medical Sciences, Tehran, Iran

**Keywords:** acupuncture, catgut embedding, evidence-based medicine, pain management, Traditional Chinese Medicine

## Abstract

Since ancient times, acupuncture has been utilized in the treatment of lots of diseases, as a part of Traditional Chinese Medicine. Acupoint embedding (AEM) therapy, known as catgut embedding, is a development of acupuncture that consists of inserting catgut or surgical threads into specific acupoints to produce continuous acupoint stimulation based on the theory of Traditional Chinese Medicine. The remaining thread in the acupoint works as a semi-permanent acupuncture needle that results in decreasing the total time of the treatment which is required for continuous manual acupuncture sessions and increasing the treatment efficacy and patients’ satisfaction. In each session of AEM about twenty 1 to 2 cm-long threads—natural origin, synthetic polymer, or bioactive threads—will be inserted at the target acupoints and this process will be repeated every 3 to 4 weeks. Indications of AEM are somehow similar to manual acupuncture including obesity, pain, musculoskeletal inflammations, infertility, etc, and it cannot be performed on pregnant women and pediatrics. AEM demonstrates its therapeutic effects via modulating immune system function, alleviating body inflammatory conditions, affecting the neurohormonal system, and other mechanisms. Subcutaneous indurations, redness, bleeding, hematoma, and bruising are some adverse events reported following the AEM. In conclusion, the scientific literature suggests that AEM is a relatively safe and convenient therapy if performed by a professional skilled practitioner.

## 1. Introduction

Acupuncture, as a part of Traditional Chinese Medicine (TCM), is a system of nonpharmacological treatment that is gaining the attention of patients and physicians all over the world.^[1]^ From several thousand years ago, acupuncture has Chinese Traditional physicians practiced acupuncture and since 16th century, it has been distributed from Eastern countries to Europe and the United States.^[2]^ Based on the principle of meridian theory, acupuncture is defined as applying thin needles to specific points, so-called acupoint, in different parts of the body, from feet to head.^[1]^ Under the ideology of TCM, health is not merely the absence of being sick, the foundation of wellness comes from the balance of Qi which is explained as “vital energy” that provides the physiological function of organs.^[3]^ Although acupuncture has become popular worldwide, some patients and healthcare professionals still have little information about the different methods, therapeutic effects, indications, cautions, safety, etc, of acupuncture.^[4]^ As stated in a cross-sectional study on the use of acupuncture in the United States, pain relief (such as lower back pain, shoulder pain, or headache) was the most common cause to refer to an acupuncturist. Other problems included mental health and mood disorders (anxiety, depression, or insomnia), musculoskeletal problems (frozen shoulder, arthritis), immune system (allergies or general enhancement), gynecology (infertility), and gastrointestinal dysfunctions (constipation).^[3]^ Over the years, different methods and approaches for acupuncture treatments have been raised, such as herbal acupuncture, electroacupuncture, laser acupuncture, or embedding. Acupoint embedding (AEM) therapy, a developed subtype of acupuncture, is a therapeutic method of inserting catgut or surgical threads into specific acupoints to produce continuous acupoint stimulation.^[5]^ Recent studies reported various applications of AEM under the guidance of TCM such as menopause, urticaria, depression, obesity, acute and chronic pain, and other health issues with limited side effects.^[1,5,6]^ According to the growing tendency to the use of AEM and the lack of a practical comprehensive text on AEM, here we aimed to conduct a brief literature survey on different features of AEM including methods, similarities and differences with manual acupuncture (MA), indication, cautions, and contraindication, mechanism of action, and side effects.

## 2. Methods and thread types

Embedding consists of inserting small pieces of absorbable threads in the location of particular responsible acupoints in accord with the patients’ problems and syndrome differentiation. The implanted thread will produce a regional inflammation stimulating the acupoint like a remaining inserted needle.^[7]^ In each session of AEM about twenty 1 to 2 cm-long sterile threads will be inserted into the tip of a disposable stainless steel sterile 20 to 21-gauge needle, implanting the needle into the target acupoints after local disinfection. The specialist pushes the thread into the site by a blunt acupuncture needle and then withdraws the needle, leaving the thread at the acupoint. To reach a satisfying outcome, this procedure needs to be repeated every 3 to 4 weeks at least for 2 months.^[8]^ Although AEM has been originated and developed in China, by attending other countries such as South Korea, Mexico, and Indonesia it was expected to develop different methods and indications; however, our literature review showed no significant difference between method of performing AEM in different countries.^[9–12]^ Generally, 3 types of embedding threads are available: natural threads including collagen or chitosan threads, synthetic polymer threads including polylactic acid-glycolic acid or polydioxanone threads, and bioactive threads including antimicrobial, silver nanoparticle, or sustained-release threads.

1. Natural threads: collagen threads mainly include sheep catgut made of collagen extracted from the small intestine of sheep mucosa. According to its high prevalent of allergic reaction and poor flexibility, catgut is usually treated with chromic acid.^[13]^ Besides, scientists developed 4 types of drug catguts according to different syndromes: clearing heat and resuscitation; promoting blood circulation and relieving blood stasis; enhancing Qi and blood; and nourishing yin and kidney.^[14]^ On the other hand, chitosan is produced by deacetylating chitin widely utilized in biomedical materials, for example, absorbable sutures without immunogenicity. The shell of different types of shrimp, crabs, and insects are rich in chitin, a natural organic polymer that contains nitrogen.^[13]^ Study of Wang WQ et al^[15]^ showed that using chitosan compared to catgut embedding results in less side effects with higher therapeutic effects.
2. Synthetic threads: polylactic acid-glycolic acid (PLGA), synthesized from lactic acid and glycolic acid is a useful polymer in biomedical engineering for example, in producing tissue repair materials or bioabsorbable sutures. The required materials for preparing PLGA are mainly derived from plants and its degradation in the human body depends on the hydrolysis of ester bonds resulting in production of small lactic acid and glycolic acid molecules that can be excreted into urine. On the other hand, polydioxanone suture fiber is produced by polymerization of *p*-dioxanone with high biocompatibility. Compared to PLGA or polydioxanone suture, despite no remarkable difference in efficacy, using sheep catgut embedding shows much more adverse events (AEs) such as tenderness, induration, local redness, and swelling.^[13]^
3. Bioactive threads: to avoid postoperative infection, antimicrobial threads have been prepared by the addition of antibacterial agents, to the absorbable thread. Embedding threads containing triclosan as an antibacterial agent, reduce the regional bacterial colonies without any toxicity or diminishing the mechanical strength of the surgical thread. Since triclosan shows bacteriostatic effects, not bactericidal, antimicrobial peptides have attracted microbiologists.^[13]^ Moreover, developing nanofibers with endogenous antimicrobial peptides, such as 25-hydroxyvitamin D3, can stimulate toll-like receptors to activate vitamin D receptor that induces generation of antimicrobial peptide hCAP18/LL37 which plays a significant role in eliminating microorganisms by damaging the bacterial cell wall.^[16]^ Polymer threads combined with silver nanoparticle, as a natural nonantibiotic antimicrobial, provide an antiinflammatory agent that effectively promote tissue repair, too. Embedding threads are coated or impregnated with these nanoparticles and then implanted in the acupoint. Although silver nanoparticle with lesser side effects is as effective as other threads, its expensive cost may put it out of the first choice.^[17]^ Furthermore, scientists developed a sustained-release thread with a potential of modulating the degrading rate and releasing the drug. This mono- or multi- filament consists of 2 parts: the core vehicle that carries the bioactive constituents and the shell which is biodegradable. Regulating the degradation rate of the thread, enhances the effectiveness of AEM, and the sustained-release drugs, based on the patients’ condition, achieve medical purposes, as well, only by a single treatment.^[13,18]^

## 3. Similarities and differences of AEM with MA

The main subjects of AEM are patients who have time limitation and cannot attend regularly acupuncture sessions (e.g., 2–3 times 1 week), due to any disability, transportation is difficult (such as elder, bedridden, or wheelchair-bound patients), or have no easy local access to a professional physician who knows acupuncture well. Moreover, medical tourists who don’t stay long time at 1 place, prefer AEM, more than MA which requires continuous treatment sessions. Several comparative studies have investigated the curative effects and safety of MA and AEM. Although the results showed both of these interventions can achieve clinical aims, in most of the studies AEM was superior to MA.^[9,19,20]^ Recent evaluations revealed that both MA and AEM may lead to tenderness, redness, bleeding, or pain, more dominant in AEM as a minimally invasive intervention; however, statistical analysis lack to conclude a certain result.^[2,19]^

## 4. Indications and contraindications

Indications and contraindications of AEM are somehow same as MA. Hence, if anywhere MA works, AEM works too, as a semi-permanent acupuncture. Despite a psychological component of the treatment is that all acupuncture methods including AEM, or even sham acupuncture, make a good feeling for the patient, same as other treatment modalities, effectiveness of the AEM is correlated to the knowledge and skill of the physician.^[1]^

The main therapeutic purposes of AEM are for management of obesity,^[7,21]^ pain,^[5]^ and any inflammation^[22]^ in musculoskeletal system (such as low back pain),^[23]^ gastrointestinal system^[6]^ (such as peptic ulcer), or obstetrics and gynecology (such as infertility^[24]^ or menopause problems^[25]^). Nowadays, most of the patients seek for AEM for the treatment of the obesity.^[26]^

On the other hand, same as MA, AEM has few restrictions. AEM cannot be performed in vulnerable patients such as pregnant women and pediatrics. Although anticoagulation is not a contraindication for AEM, it should be considered. It is better not to do AEM on infectious site of the skin or any cutaneous problems such as malignancies. Also, it is not recommended to do AEM for patients with psychos or delusions.^[1]^

As the present study is based on the Chinese concept of AEM, we briefly summarize available evidence published by researchers of Korea, Mexico, and Indonesia. A Korean survey on the effects of AEM on neck pain showed that AEM alleviated the pain, reduced disability index scores, and showed higher efficacy rate compared to control.^[27]^ Moreover, in patients with carpal tunnel syndrome Korean method of AEM showed remarkable effects on finger tingling, Phalen test, and pain severity.^[11]^ Mexican TCM scientists compared the effects of AEM with fluoxetine in depressed rat model and showed antidepressant effects of AEM and its potential to remodel the hippocampus.^[12]^ AEM normalized immobility behavior, the levels of testosterone, estradiol, and corticosterone hormones, and the content of brain-derived neurotrophic factor.^[28]^ Also, Garcia-Vivas et al^[9]^ conducted a randomized clinical trial in Mexico and revealed that AEM had favorable effects on the reduction of risk of diabetes by reducing body weight, regulating serum insulin level, and enhancing insulin sensitivity, as well as changes in gene expression in adipose tissue showed alterations in genes involved in the regulation of homeostasis, lipid metabolism, olfactory signal transmission, and the γ-aminobutyric acid signaling pathway.^[29]^ Furthermore, Indonesian scientists performed a study on the effect of AEM on essential hypertension. They indicated that AEM can influence the clinical effects of high systolic and diastolic blood pressure and serum level of nitric oxide.^[30]^ For management of obesity in Indonesia, patients who underwent AEM showed remarkable reduction in waist circumference, as well as serum tumor necrosis factor-α.^[10]^ Likewise, the study of Tjan et al^[31]^ showed reduction in body mass index in addition to interleukin-6. On the contrary, Fitri et al^[32]^ showed no significant change in interleukin-6 in patients with gastroesophageal reflux disease.

## 5. Mechanism of action

There have been limited studies investigating the mechanism of action behind AEM, a treatment method that combines both acupuncture and the use of suture thread. AEM is considered a mixed treatment, combining multiple approaches and producing both systemic and local effects. The insertion process for AEM is similar to acupuncture, but in AEM, the suture thread remains inside the body, providing continuous biostimulation.^[23,24]^

The systemic effects of AEM are attributed to its ability to induce prolonged stimulation. Catgut, a type of heterogeneous protein, plays a role in the local effects of acupoint catgut embedding (ACE). Once inserted into acupoints, it undergoes processes such as softening, decomposition, liquidation, and absorption, effectively promoting and enhancing nutrition metabolism. Additionally, ACE has been found to improve the body’s stress response, vascular permeability, and blood circulation.^[24]^

Previous studies have reported positive effects of AEM in various disorders and diseases. Some of these studies have also explored the mechanism of action from a modern medical perspective:

- Modulating immune system function: the use of absorbable thread implantation in acupoints during thread embedment therapy introduces a foreign protein to the body, which can stimulate allergic reactions and trigger the production of lymphatic factors. This process enhances the phagocytic function of macrophages, thereby boosting the body’s immune function.^[5]^
- Alleviating inflammation: the stimulation caused by the absorbable thread embedded in acupoints has been found to improve the body’s ability to cope with stress. It promotes local vasodilation, increases the formation of new blood vessels, enhances blood flow, improves local lymphatic and blood circulation, accelerates tissue metabolism, and consequently reduces exudative adhesions while facilitating the absorption of inflammation.^[5]^
- Pain management: AEM can stimulate the central nervous system, triggering nerve impulses and promoting the secretion and synthesis of endogenous opioid and neuropeptide substances like endorphins. This process raises the pain threshold and inhibits the release of inflammatory factors such as nitric oxide, effectively blocking the feedback loop of pain signals. Additionally, a separate study suggested that AEM exhibited antihyperalgesic effects in rats by inhibiting the Sigma-1 receptor, which modulates p38 mitogen-activated protein kinases but not extracellular signal-regulated kinase.^[5,22]^
- Alleviating muscle pain: the slow hydrolysis of the embedded thread within the tissue can act as a long-lasting stimulator for collagen fiber formation, achieved by downregulating the activity of c-Jun N-terminal kinase and matrix metalloproteinas-9. Furthermore, AEM provides significant relief from muscle pain by continuously stimulating the core muscles and ligaments.^[23]^
- Weight management: AEM has been found to help control insulin resistance and improve leptin resistance. Leptin, an adipokine that suppresses appetite and promotes energy metabolism, exhibits decreased levels in both serum and hypothalamus. Although a study by Li et al (2021) suggested that the efficacy of AEM in treating obesity may be related to altered intestinal flora, this relationship has not been confirmed by any study thus far.^[6,9,26]^
- Appetite control: AEM has been shown to activate the satiety center and inhibit the hunger center in rats by regulating norepinephrine, dopamine, 5-hydroxytryptamine (serotonin), and 5-hydroxyindoleacetic acid within the feeding center. These effects may contribute to the observed enhancements in anthropometric and biochemical parameters.^[9]^
- Prevention and treatment of osteoporosis: AEM has the potential to regulate the hypothalamic-pituitary-ovarian axis, leading to increased serum E2 levels. This mechanism may be significant in preventing and treating osteoporosis in postmenopausal women.^[25]^

## 6. Mechanism according to TCM

AEM represents an updated and improved form of traditional MA, offering advantages such as reduced cost and time.^[7,19]^ This specialized form of puncture treatment has demonstrated comparable results to classic MA but with fewer required visits. The key underlying mechanism involves the insertion of catgut or absorbable medical threads into relevant acupoints, producing a lasting, slow, and stable acupuncture effect.^[5,7,23]^

The combined effects of proteolytic enzymes and macrophage activity against the absorbable surgical thread have been reported to enhance and prolong acupoint stimulation for a period of 15 to 21 days. This occurs due to mild irritation in the subcutaneous tissue.^[9]^

In a study conducted by Pei et al,^[5]^ AEM was demonstrated as a compound treatment method that integrates multiple effects, including the acupoint sealing effect, acupuncture effect, pricking blood effect, and embedding needle effect. These effects are distinct characteristics of traditional Chinese medicine. AEM achieves its therapeutic goals through the adjustment of the viscera, regulation of blood, and balance of yin and yang. Its purpose is to remove blockages in the flow of vital energy or life force, known as “Qi,” which circulates throughout the body via a system of pathways called channels.^[5,9]^

## 7. Side effects

While AEM is commonly used in clinics and hospitals worldwide, there is a lack of comprehensive evidence regarding its safety. AEM is generally considered safe, but because it involves the insertion of a thread, the possibility of infection should be taken into account.^[23]^

A randomized controlled pilot trial by Lee et al^[23]^ examined the safety of ACE by conducting hematological and biochemical tests before treatment initiation, 2 days after the first session, and 8 weeks after treatment initiation. The results showed no signs of infection in any participant, supporting the safety of ACE over a 10-week period. However, long-term AEs of ACE still need to be studied.^[23]^

A systematic review and meta-analysis by Guo et al^[19]^ analyzed 43 studies on ACE and obesity. Among these studies, one of them reported AEs, including subcutaneous indurations (n = 5), redness, and swelling (n = 1).^[33]^ Another systematic review by Sheng et al^[7]^ found that among 15 studies, only 2 trials reported side effects, such as fainting (n = 1), subcutaneous indurations (n = 2), hematoma, and bruising (n = 2).^[21,34]^ In a systematic review by Huang et al,^[35]^ which included 61 studies (45 RCTs and 16 case reports) with 620 cases of AEs, various AEs were summarized. The most common AEs were induration, bleeding and ecchymosis, redness and swelling, fever, and pain. They were accounted for 75.35% in the review with most of them being mild. The rarest AEs included epilepsy, irregular menstruation, skin ulcer, thread malabsorption, and fat liquefaction. However, not all of these AEs had a clear causal relationship with AEM. Most AEs were local reactions, while systemic reactions were rare. Severe AEs were rarely reported, with only 5 cases (0.1%) reported from 2013 to 2017 using catgut thread, which is seldom used nowadays due to the wide use of new absorbable surgical suture. All severe AEs resolved after symptomatic treatment with no lasting effects.^[35]^

Recent reports have described tender, erythematous, subcutaneous nodules at the site of the catgut embedding, which were considered foreign-body reactions. Because of protein nature of ACE, the body recognizes the thread as a foreign protein; hence, AEs may occur, including increased temperature of the region, redness, rash, or itching may occur at the embedding site. In addition, since the body state of patients are different from each other, some patients may experience nodules, cysts, or a local mass.^[13]^ The thread used in AEM is mostly PDA, with less consideration of foreign-body reactions; therefore, AEM appears to be safer than ACE in terms of foreign-body reactions.^[23]^

## 8. Conclusion

The evidence suggests that AEM is a relatively safe and convenient therapy, particularly with the use of new absorbable surgical sutures. Improving practitioner skills, adhering to proper procedures, and monitoring patient conditions can contribute to reducing the incidence of AEs and enhancing the safety of AEM.

## Author contributions

**Data curation:** Reihane Alipour, Moein Jamali Dastjerdi, Mohammad Sadegh Adel-Mehraban.

**Investigation:** Reihane Alipour, Mohammad Sadegh Adel-Mehraban.

**Writing—original draft:** Reihane Alipour, Moein Jamali Dastjerdi, Mohammad Sadegh Adel-Mehraban.

**Writing—review & editing:** Reihane Alipour, Moein Jamali Dastjerdi, Mohammad Sadegh Adel-Mehraban, Amir Hooman Kazemi.

**Project administration:** Mohammad Sadegh Adel-Mehraban.

**Conceptualization:** Amir Hooman Kazemi.

**Methodology:** Amir Hooman Kazemi.

**Supervision:** Amir Hooman Kazemi, Reihane Alipour.

**Validation:** Amir Hooman Kazemi, Reihane Alipour.
